# Multiparametric imaging using ^18^F-FDG PET/CT heterogeneity parameters and functional MRI techniques: prognostic significance in patients with primary advanced oropharyngeal or hypopharyngeal squamous cell carcinoma treated with chemoradiotherapy

**DOI:** 10.18632/oncotarget.15904

**Published:** 2017-03-04

**Authors:** Sheng-Chieh Chan, Nai-Ming Cheng, Chia-Hsun Hsieh, Shu-Hang Ng, Chien-Yu Lin, Tzu-Chen Yen, Cheng-Lung Hsu, Hung-Ming Wan, Chun-Ta Liao, Kai-Ping Chang, Jiun-Jie Wang

**Affiliations:** ^1^ Department of Nuclear Medicine, Keelung Chang Gung Memorial Hospital, Keelung, Taiwan; ^2^ Molecular Imaging Center, Linkou Chang Gung Memorial Hospital and Chang Gung University, Taoyuan, Taiwan; ^3^ Department of Nuclear Medicine, Linkou Chang Gung Memorial Hospital and Chang Gung University, Taoyuan, Taiwan; ^4^ Department of Internal Medicine, Division of Medical Oncology, Linkou Chang Gung Memorial Hospital and Chang Gung University, Taoyuan, Taiwan; ^5^ Department of Diagnostic Radiology, Linkou Chang Gung Memorial Hospital and Chang Gung University, Taoyuan, Taiwan; ^6^ Department of Medical Imaging and Radiological Sciences, Chang Gung University, Taoyuan, Taiwan; ^7^ Department of Radiation Oncology, Linkou Chang Gung Memorial Hospital and Chang Gung University, Taoyuan, Taiwan; ^8^ Department of Otorhinolaryngology, Linkou Chang Gung Memorial Hospital and Chang Gung University, Taoyuan, Taiwan

**Keywords:** PET, heterogeneity, DCE-MRI, DWI, head and neck cancer

## Abstract

**Background:**

In this study, PET heterogeneity was combined with functional MRI techniques to refine the prediction of prognosis in patients with oropharyngeal or hypopharyngeal squamous cell carcinoma (OHSCC).

**Methods:**

A total of 124 patients with primary advanced OHSCC who underwent pretreatment ^18^F-FDG PET/CT, dynamic contrast-enhanced MR imaging (DCE-MRI), and diffusion-weighted MR imaging (DWI) were enrolled. Conventional and heterogeneity parameters from ^18^F-FDG PET as well as perfusion parameters from DCE-MRI and diffusion parameter from DWI of primary tumors were analyzed in relation to recurrence-free survival (RFS) and overall survival (OS).

**Results:**

Multivariate analysis identified hypopharyngeal tumors (*P* = 0.038), alcohol drinking (*P* = 0.006), K^*trans*^ ≤ 0.5512 (*P* = 0.017), and K_*ep*_ ≤ 0.8872 (*P* = 0.005) as adverse prognostic factors for RFS. Smoking (*p* = 0.009), K^*trans*^ ≤ 0.5512 (*P* = 0.0002), K_*ep*_ ≤ 0.8872 (*P* = 0.004), and the PET heterogeneity parameter uniformity ≤ 0.00381 (*P* = 0.028) were independent predictors of poor OS. The combination of PET uniformity with DCE-MRI parameters and smoking allowed distinguishing four prognostic groups, with 3-year OS rates of 100%, 76.6%, 57.4%, and 7.1%, respectively (*P* < 0.0001). This prognostic system appeared superior to both the TNM staging system (*P* = 0.186) and the combination of conventional PET parameters with DCE-MRI (*P* = 0.004).

**Conclusions:**

Multiparametric imaging based on PET heterogeneity and DCE-MRI parameters combined with clinical risk factors is superior to the concomitant use of functional MRI coupled with conventional PET parameters. This approach may improve the prognostic stratification of OHSCC patients.

## INTRODUCTION

Head and neck malignancies are a heterogeneous group of cancers arising within the upper aerodigestive tract that collectively represent the seventh most commonly diagnosed cancer worldwide [1]. Histologically, most head and neck malignancies are squamous cell carcinomas. Primary oropharyngeal and hypopharyngeal squamous cell carcinomas (OHSCC) are distinct among head and neck cancers in terms of etiology, lymphatic drainage from adjacent organs, and treatment approach.

Owing to the rich lymphatic supply and soft tissue boundaries of the oropharynx and hypopharynx, most patients with OHSCC present with locoregionally aggressive tumors and advanced disease at presentation. In the absence of distant metastases, chemoradiotherapy is currently considered the mainstay of treatment for OHSCC [2]. However, current treatment outcomes remain suboptimal, especially in presence of locoregionally advanced disease. In addition, up to 26% of patients who achieve a clinical complete response after chemoradiotherapy still have evidence of residual regional lymph node metastases [3]. In this scenario, there is an urgent need to improve prognostic stratification, with the ultimate goal of optimizing follow-up schedules, treatment planning, and clinical outcomes.

Interest in multiparametric imaging to investigate the biological properties of malignant tumors is increasing. Dynamic contrast-enhanced MRI (DCE-MRI) and diffusion-weighted MR imaging (DWI) have been shown to be clinically useful for predicting treatment responses and identifying disease relapses in patients with head and neck tumors [4-14]. DWI detects the diffusion of water molecules in tissues that may reflect tumor cellularity. DCE-MRI can probe the tumor microvascular environment, including perfusion, vessel permeability, and volume of extracellular space [15]. The use of CT perfusion in the assessment of tumor vascular physiology and hemodynamic has recently gained momentum. CT-perfusion parameters − including blood volume, blood flow, mean transit time, and permeability surface product − have been shown to be significantly associated with tumor response to radiotherapy and chemotherapy in patients with head and neck cancer [16-18]. Differently from the logarithmic relation between signal intensity and the concentration of paramagnetic contrast medium observed in DCE-MRI, the main advantage of CT perfusion lies in the linear relationship between the concentration of contrast medium and CT attenuation, which facilitates a quantitative measurement of perfusion parameters [19]. Nonetheless, CT perfusion is not without disadvantages - including radiation exposure and the risk of adverse reactions to the iodinated contrast medium (e.g., contrast-induced nephropathy, anaphylactic shock, and bronchospasm).

^18^F-fluorodeoxyglucose-positron emission tomography (^18^F-FDG PET)/CT is increasingly being used in the pretreatment evaluation and prognostic stratification of patients with head and neck squamous cell carcinoma (HNSCC) [20-25]. Although the combination of DWI, DCE, and PET in a multiparametric manner can help refining the prognostic prediction, only a few studies in OHSCC patients have been conducted to date [6, 10, 26]. Moreover, the available reports were based on conventional PET parameters - including standardized uptake value (SUV), metabolic tumor volume (MTV), and total lesion glycolysis (TLG) - that provide a general measure of tumor glucose metabolism. Unfortunately, these parameters are unable to capture the heterogeneity of intratumor FDG distribution, a promising prognostic feature of most solid malignancies. Intratumor diversity has significant implications for both the prognosis and therapeutic response of cancer patients, with tumor heterogeneity as an imaging biomarker being generally associated with aggressive and less treatment sensitive malignancies [27].

Measures of spatial heterogeneity on ^18^F-FDG PET images have been shown to predict prognosis in cancer patients, including those with head and neck carcinoma [28-31]. However, it is still unclear whether the combination of ^18^F-FDG PET/CT heterogeneity, DCE-MRI, and DWI parameters can improve the prognostic stratification of patients with advanced OHSCC. We therefore designed the current study to examine this issue in a patient population homogeneously treated with chemoradiotherapy.

## RESULTS

Between January 2010 and May 2013, we identified 149 OHSCC patients who were potentially eligible for the study. However, patients with unsatisfactory DWI or DCE-MRI images (n = 20) and loss to follow-up (n = 5) were excluded. Consequently, the final study cohort consisted of 124 patients (63 with cancer of the oropharynx and 61 with cancer of the hypopharynx; Table 1). The median follow-up times in the entire study cohort and surviving patients were 28.7 months (range, 2-58 months) and 36 months (range, 17-58 months), respectively. Of the 124 study patients, 29 experienced local failure, 32 regional nodal metastasis, and 20 distant failure. At the time of analysis, 47 (37 %) patients were dead. The median RFS was 22 months (range, 2-58 months). The 3-year RFS and OS rates were 76.1% and 62.2%, respectively.

**Table 1:** General characteristics of the study patients

Characteristic	*N*
Age, years (mean ± standard deviation)	52 ± 10
Sex	
Male	116
Female	8
Tumor subsite	
Oropharynx	63
Hypopharynx	61
T status	
T1	5
T2	26
T3	20
T4a	65
T4b	8
N status	
N0	15
N1	12
N2b	60
N2c	22
N3	15
Disease stage	
III	9
IVA	87
IVB	28
Smoking	
Yes	94
No	30
Alcohol drinking	
Yes	80
No	44
Betel quid chewing	
Yes	62
No	62

Data are given as counts unless otherwise indicated.

### Predictors of survival endpoints

**Figure 1:**
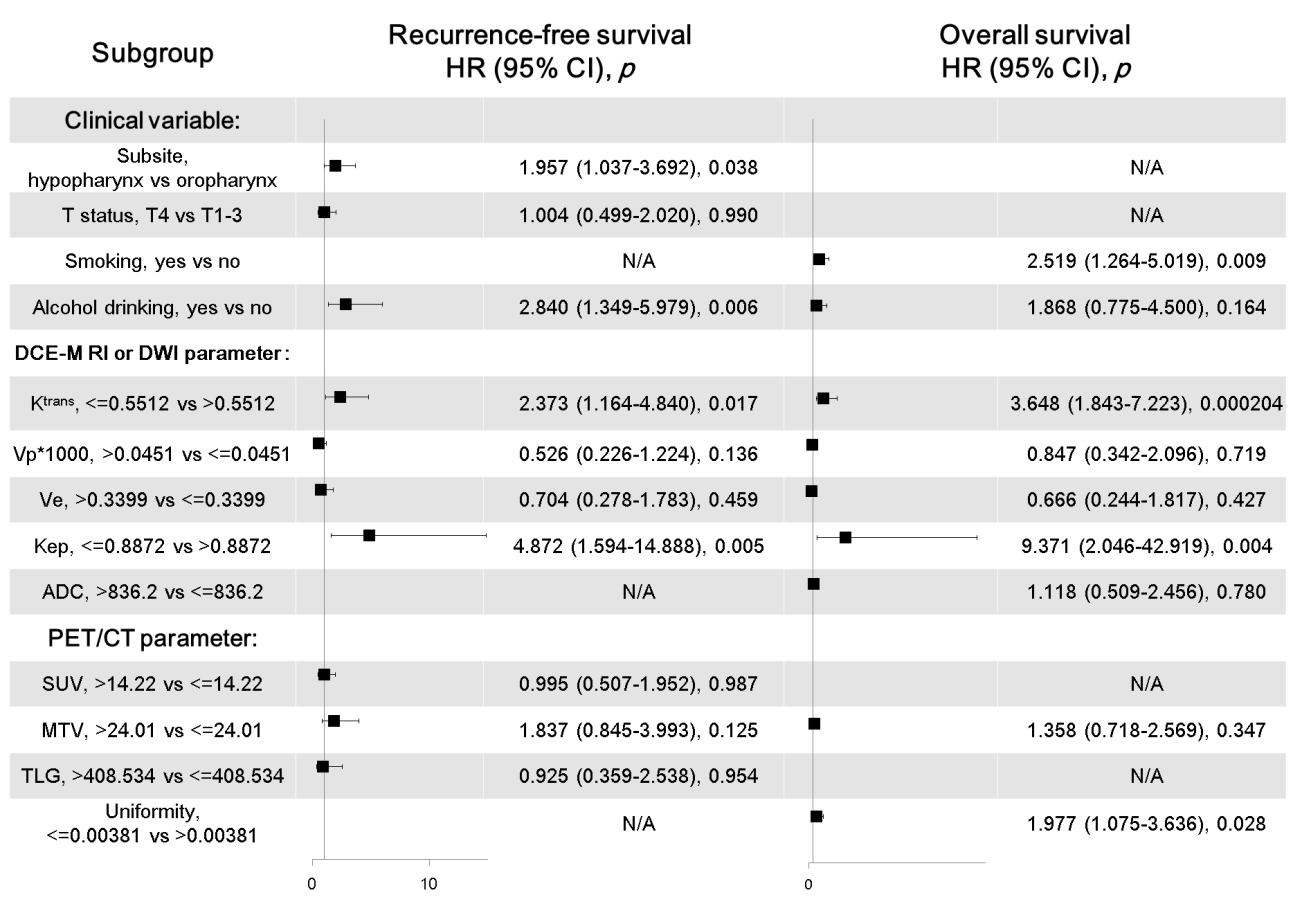
Multivariate Cox regression analyses of recurrence-free survival and overall survival in OHSCC patients Clinical variables, DCE-MRI and DWI parameters, as well as PET/CT parameters were included as covariates in the multivariate model. The results indicated that tumor subsite, alcohol drinking, K^*trans*^, and K_*ep*_ were independent predictors of recurrence-free survival, whereas smoking, K^*trans*^, K_*ep*_, and uniformity independently predicted overall survival. CI, confidence interval; HR, hazard ratio; N/A, not applicable.

Univariate analysis identified the following parameters as significant predictors of RFS: tumor subsite, T status, alcohol drinking, K^*trans*^, V_*p*_, V_*e*_, K_*ep*_, SUV, MTV, TLG, heterogeneity parameters entropy, contrast, busyness, and complexity (Tables 2 and 3). Because different PET heterogeneity parameters were characterized by a high degree of collinearity, each of them was entered separately into the multivariate Cox regression model. After adjustment for potential confounders, multivariate analysis identified hypopharyngeal tumors (*P* = 0.038), alcohol drinking (*P* = 0.006), K^*trans*^ ≤ 0.5512 (*P* = 0.017), and K_*ep*_ ≤ 0.8872 (*P* = 0.005) as adverse prognostic factors for RFS (Figure 1). Smoking, alcohol drinking, K^*trans*^, V_*e*_, V_*p*_, K_*ep*_, ADC, MTV, and most heterogeneity parameters were significantly associated with OS in univariate analysis (Tables 2 and 3). However, only smoking, K^*trans*^, K_*ep*_, and the heterogeneity parameter uniformity retained their independent prognostic significance for OS in multivariate analysis (Figure 1).

**Table 2:** Univariate analyses of 3-year recurrence-free survival and overall survival rates in primary OHSCC patients

Risk factors (N)	3-year recurrence-free survival		3-year overall survival	
	**% (*N* of events)**	***P* value**	**% (*N* of events)**	***P* value**
Age (years)		0.081		0.226
≤ 50 (63)	47.7 (31)		43.4 (28)	
> 50 (61)	65.9 (19)		63.4 (19)	
Sex		0.157		0.501
Male (116)	55.0 (49)		49.1 (45)	
Female (8)	83.3 (1)		75.0 (2)	
Tumor subsite		0.006		0.656
Oropharynx (63)	68.4 (18)		41.2 (25)	
Hypopharynx (61)	44.6 (32)		56.6 (22)	
Hemoglobin (g/dL)		0.262		0.390
≤ 14.3 (73)	49.7 (24)		41.8 (22)	
> 14.3 (51)	61.4 (26)		59.2 (25)	
T status		0.038		0.093
T1-3 (51)	67.4 (15)		52.2 (15)	
T4 (73)	49.2 (35)		48.2 (32)	
N status		0.126		0.646
N0-1 (27)	67.3 (7)		62.9(9)	
N2-3 (97)	53.6 (43)		47.5(38)	
Disease stage		0.136		0.186
III (9)	87.5 (1)		64.8 (3)	
IVA (87)	57.4 (35)		56.2 (29)	
IVB (28)	46.5 (14)		37.4 (15)	
Smoking		0.095		0.003
Yes (94)	47.2 (40)		47.8 (45)	
No (30)	69.2 (2)		82.9 (5)	
Alcohol drinking		0.041		0.003
Yes (80)	44.8 (36)		44.4 (40)	
No (44)	65.8 (11)		76.9 (10)	
Betel quid chewing		0.797		0.150
Yes (62)	52.8 (23)		47.2 (29)	
No (62)	52.0 (24)		65.5 (21)	
Ktrans (min−1)		0.003		0.025
≤ 0.551 (68)	49.2 (33)		39.9 (33)	
> 0.551 (56)	66.2 (17)		62.9 (14)	
Vp*1000		0.001		0.004
≤ 0.045 (24)	56.6 (10)		62.6 (7)	
> 0.045 (100)	56.9 (40)		49.4 (40)	
Ve		0.027		0.038
≤ 0.339 (98)	52.0 (44)		45.6 (42)	
> 0.339 (26)	75.1 (6)		69.8 (5)	
Kep (min−1)		0.035		0.02
> 0.887 (20)	70.2 (5)		89.4 (2)	
≤ 0.887 (104)	53.5 (45)		57.8 (45)	
ADC (× 10-3 mm2/s)		0.695		0.045
≤ 836.2 (22)	80.4 (4)		58.3 (8)	
> 836.2 (102)	51.3 (46)		49.1 (39)	
SUV (g/mL)		0.032		0.176
≤ 14.22 (54)	60.5 (19)		59.7 (15)	
> 14.22 (70)	53.8 (31)		43.9 (32)	
MTV (mL)		0.007		0.023
≤ 24.01 (80)	61.2 (29)		56.8 (35)	
> 24.01 (44)	48.2 (21)		40.4 (22)	
TLG (g/mL × mL)		0.039		0.345
≤ 408.534 (111)	58.2 (43)		52.0 (40)	
> 408.534 (13)	43.1 (7)		38.9 (7)	

Abbreviations: ADC, apparent diffusion coefficient; SUV, standardized uptake value; MTV, metabolic tumor volume; TLG, total lesion glycolysis.

**Table 3:** Univariate analysis of heterogeneity parameters in relation to overall survival and recurrence-free survival

Heterogeneity parameter	Cut-off for RFS	Cut-off for OS	RFS	OS
			***P* value**	***P* value**
**Second-order texture features or heterogeneity parameters based on NGLCM**				
Uniformity (32 bins)	0.004	0.004 (0.00396)	NS	0.01
Contrast (32 bins)	46.495	46.495	NS	NS
Entropy (32 bins)	5.785	5.785	NS	0.047
Homogeneity (32 bins)	0.276	0.276	NS	NS
Dissimilarity (32 bins)	5.275	5.275	NS	NS
Inverse difference moment (32 bins)	0.170	0.170	NS	0.041
Uniformity (64 bins)	0.001	0.001	NS	0.044
Contrast (64 bins)	173.88	173.88	NS	NS
Entropy (64 bins)	6.939	6.939	0.04	0.02
Homogeneity (64 bins)	0.179	0.179	NS	0.03
Dissimilarity (64 bins)	9.71	9.71	NS	NS
Inverse difference moment (64 bins)	0.09	0.09	NS	0.025
**Higher-order texture features or heterogeneity parameters based on NGTDM**				
Coarseness (32 bins)	0.006	0.006	NS	0.023
Contrast (32 bins)	0.001	0.001	NS	0.027
Busyness (32 bins)	1.668	1.668	NS	0.045
Complexity (32 bins)	6.371	6.371	NS	0.036
Strength (32 bins)	5.117	5.117	NS	0.032
Coarseness (64 bins)	0.006	0.006	NS	0.023
Contrast (64 bins)	0.038	0.038	0.022	NS
Busyness (64 bins)	0.177	0.177	0.041	NS
Complexity (64 bins)	3.160	31.494	0.049	0.027
Strength (64 bins)	17.55	17.55	NS	0.019

Abbreviations: OS, overall survival; RFS, recurrence-free survival; NGLCM, normalized gray-level co-occurrence matrix; NGTDM, neighborhood gray-tone difference matrix; NS, not significant.

sNSNSNGTDM, neighborhood gray-tone difference matrix; NS, non-significant.

We then formulated a prognostic scoring system by summing up the independent prognostic factors identified in multivariate analysis for each survival endpoint. Specifically, they included smoking status, K^*trans*^, K_*ep*_, and PET uniformity for OS, and tumor subsite, alcohol drinking status, K^*trans*^, and K_*ep*_ for RFS. Each risk factor was assigned a value of 1 when present or 0 when absent (total score ranging from 0 to 4). All of the study patients carried at least one independent risk factor. The prognostic scoring system allowed stratifying patients into different risk groups with distinct 3-year OS rates (100% for patients with a score of 1, 76.6% for patients with a score of 2, 57.4% for patients with a score of 3, and 7.1% for patients with a score of 4, respectively, P < 0.0001) and 3-year RFS rates (89.7% for patients with a score of 1, 70.1% for patients with a score of 2, 45.7% for patients with a score of 3, and 14.3% for patients with a score of 4, respectively, P < 0.0001). Fifteen patients with a score of 4 (i.e., carrying all of the independent risk factors) showed significantly worse control and survival rates − including a high relapse rate (12/15, 80%) − and generally died within 3 years after the initial treatment. Patients with a score of 1 had good control and survival rates. Notably, only 10% of them (2/20) showed tumor relapse and none died after a median period of 22 months. In multivariate Cox proportional hazard analysis, patients with a score of 3 showed a better OS (hazard ratio [HR] = 0.229, *P* < 0.0001) than those with a score of 4 (reference category). Patients with scores of 2 (HR = 0.130) or 1 (HR = 0.008) similarly showed better OS rates.

**Figure 2:**
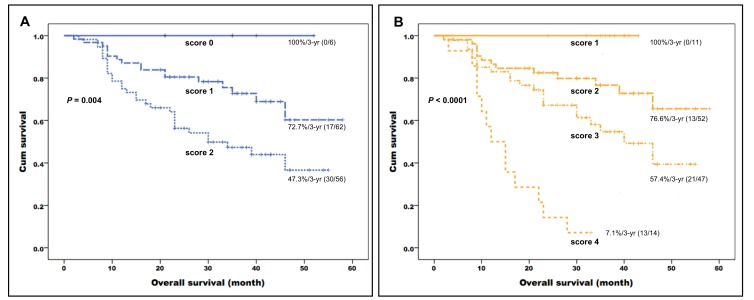
Kaplan-Meier plots of overall survival in patients with primary oropharyngeal or hypopharyngeal carcinoma stratified according to the independent risk factors identified in the previous cohort (K_ep_ and SUV of the primary tumor**; A.)** and the current study (smoking, K^*trans*^, K_*ep*_, uniformity of the primary tumor; B.). The combination of clinical variables, DCE-MRI, and PET heterogeneity parameters identified in the current study enabled a better prognostic stratification than that provided by the previous model (*P* < 0.0001 *versus* 0.004, respectively).

**Figure 3:**
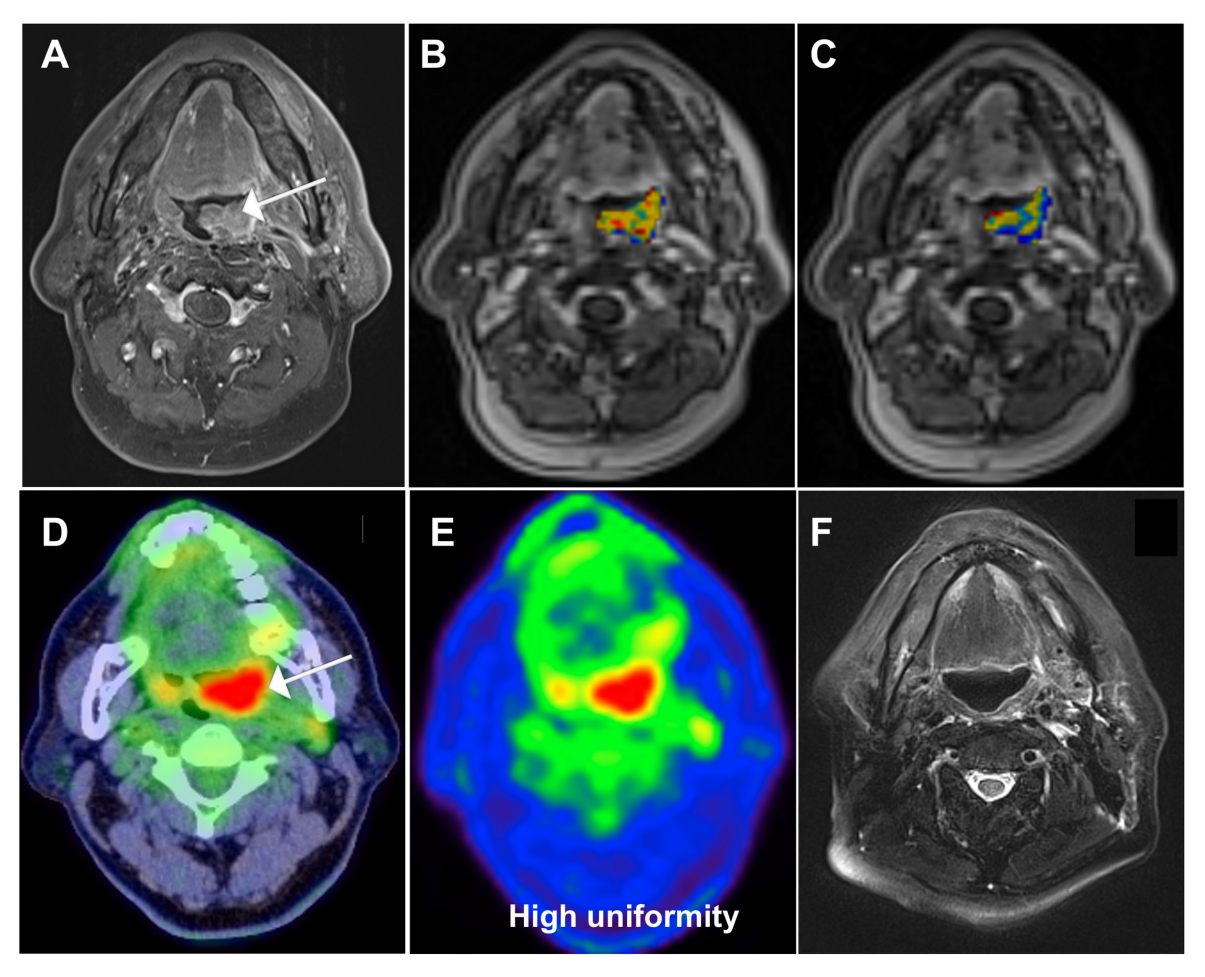
A 62-year-old male patient with oropharyngeal squamous cell carcinoma and a score of 1 based on the prognostic system developed in the current study **A.** Pretreatment axial-enhanced MRI image showing a left oropharyngeal tumor (arrow). **B.** The corresponding DCE-MRI image with an overlaid K^*trans*^ map of the primary tumor showed a value of 1.056 min^−1^. **C.** The corresponding DCE-MRI image with an overlaid K_*ep*_ map of the primary tumor showed a value of 1.1356 min^−1^. **D.**, **E.** The corresponding ^18^F-FDG PET/CT image showed a uniformity value of 0.0047 and a SUV of 15.61 g/mL. **F.** Post-treatment axial-enhanced MRI showed complete regression of the primary tumor. After 41 months of follow-up, the patients remained disease-free. Of note, this case would have been classified as having a poorer outcome (with a score of 2) using the modified prognostic scoring system reported in the previous study (Table 3).

**Figure 4:**
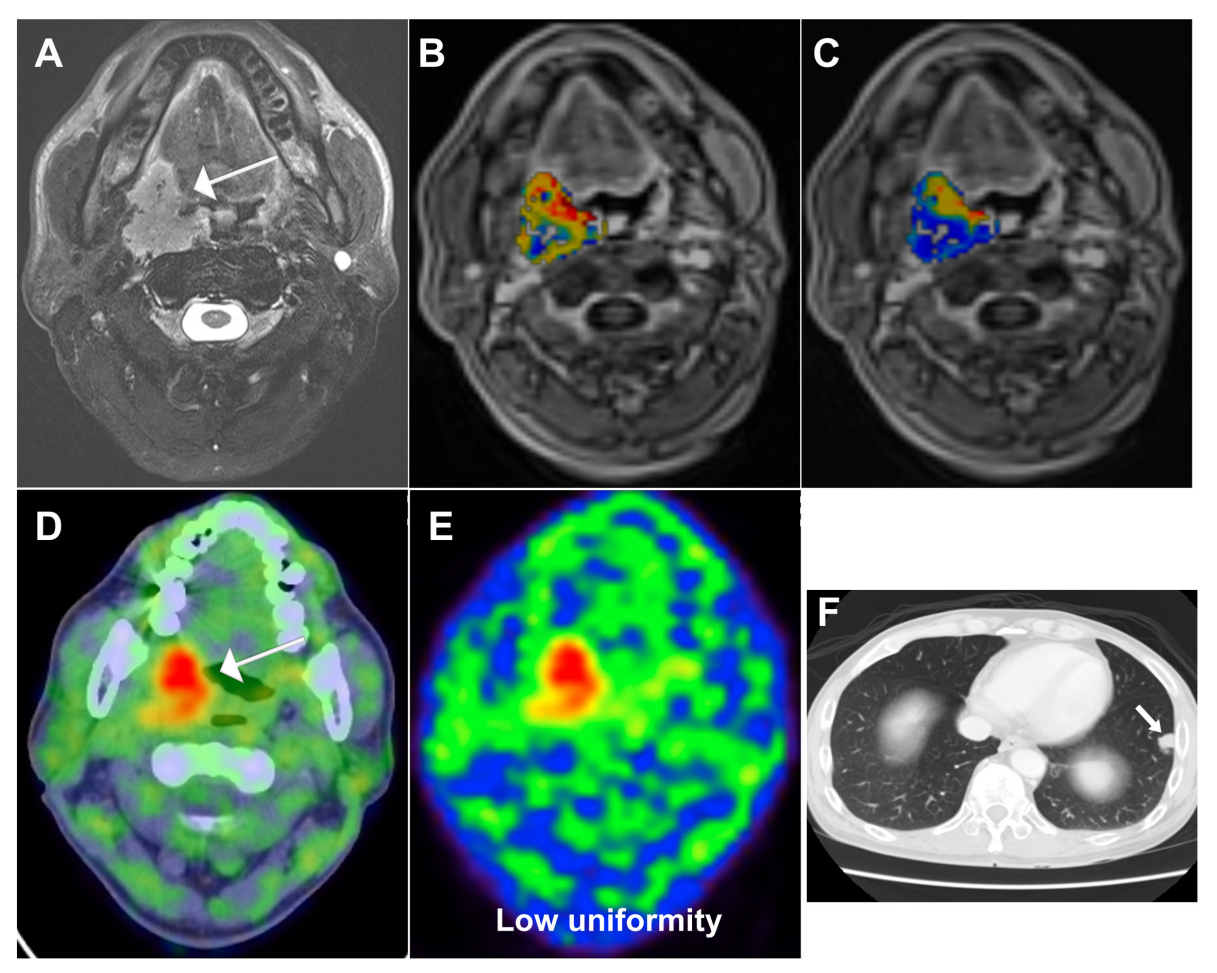
A 51-year-old male patient with oropharyngeal squamous cell carcinoma and a score of 4 based on the prognostic system developed in the current study **A.** Pretreatment axial-enhanced MRI image showing a right oropharyngeal tumor (arrow). **B.** The corresponding DCE-MRI image with an overlaid K^*trans*^ map of the primary tumour showed a value of 0.5116 min^−1^. **C.** The corresponding DCE-MRI image with an overlaid K_*ep*_ map of the primary tumor showed a value of 1.3648 min^−1^. **D.**, **E.** The corresponding ^18^F-FDG PET/CT image showed a uniformity value of 0.0032 and a SUV of 8.92 g/mL. **F.** The patient developed a distant recurrence to the lung and died 10 months thereafter. Of note, this case would have been classified as having a favorable outcome (with a score of 1) using the modified prognostic scoring system reported in the previous study (Table 3).

We then compared our current prognostic stratification system with a scoring method described in a previous paper from our group [26], based on conventional PET and functional MRI parameters. The system developed in the current study allowed a better stratification of OS (*P* < 0.0001) compared with the modified model derived from the previously published research [26] (*P* = 0.004, Figure 2). Representative images of patients with different scores are shown in Figures 3 and 4.

Table 4 shows the comparative results of the two prognostic systems in the prediction of OS. As far as the current stratification system is concerned, patients with lower scores (1−3) had significantly better survival rates and their HRs were significantly lower than those with a score of 4. In contrast, the use of the system derived from previous research failed to produce such distinct survival patterns as well as clear differences in HRs.

**Table 4:** Comparison of overall survival rates according to the prognostic stratification system devised in the current study versus that reported in the previously published research [26]*

Stratification according to the current study (risk factors: smoking, K^*trans*^, K_*ep*_, uniformity of the primary tumor)			Stratification according to previously published research [26]* (risk factors: K_*ep*_, SUV of the primary tumor)		
	***P* value**	**HR (95% CI)**		***P* value**	**HR (95% CI)**
Score 1 (*n* = 11)	0.041	0.008 (0−0.825)	Score 0 (*n* = 6)	0.198	0.041 (0−5.289)
Score 2 (*n* = 52)	<0.0001	0.130 (0.056−0.301)	Score 1 (*n* = 62)	0.008	0.444 (0.245−0.806)
Score 3 (*n* = 47)	<0.0001	0.229 (0.109−0.480)	Score 2 (*n* = 56)	Reference	Reference
Score 4 (*n* = 14)	Reference	Reference			

HR: hazard ratio; CI: confidence interval. *Compared with reference [26], this prognostic stratification system was modified to comprise only K*ep* and SUV of the primary tumor (i.e., with the exclusion of the nodal relative volume of extracellular extravascular space [V*e*-node]).

### Association between clinical, functional MRI, and heterogeneity PET parameters

Supplementary Table 2 summarizes the associations between the clinicopathological characteristics, functional MRI variables, and heterogeneity PET parameters identified as independent prognostic factors in multivariate analysis. Uniformity was significantly associated with both T classification and tumor stage. Only modest associations between functional MRI parameters and heterogeneity PET variables were identified in correlation analyses.

### Results obtained when the adaptive threshold method was applied

Besides a fixed SUV threshold of 2.5, we also tested an adaptive threshold method for tumor segmentation. When this approach was applied, we identified alcohol drinking, K^*trans*^, K_*ep*_, and uniformity as independent risk factors for overall survival in multivariate analysis (Supplementary Table 4). When a scoring system was formulated using these four independent risk factors, a similar stratification of survival curves was evident. However, the survival differences were less pronounced compared with the scoring system obtained when a fixed SUV threshold of 2.5 was used (Supplementary Figure 1). As far as recurrence-free survival is concerned, the results obtained with the two segmentation methods were similar (Supplementary Table 5).

## DISCUSSION

Most solid malignancies are characterized by substantial intratumor heterogeneity in terms of metabolism, vascularity, cellular proliferation, or biological characteristics [28-31, 33]. The results of the current study demonstrate that imaging heterogeneity on 18F-FDG PET scans is associated with survival rates in patients with primary OHSCC. Most importantly, we show that its combination with DCE-MRI parameters may allow a better prognostic stratification compared with the traditional TNM staging system or even the combination of the conventional PET parameters and functional MRI measures.

Recent studies suggested that PET heterogeneity parameters are more informative than conventional parameters such as SUV [29, 32-34] for the prediction of prognosis and treatment outcomes in patients with malignancies. In the current report, we demonstrate that heterogeneity on PET scans is prognostically superior to traditional parameters (i.e., SUV, MTV, and TLG) in OHSCC patients. This observation can be explained by the fact that conventional PET parameters are unable to capture tumor metabolic heterogeneity. In a mouse model of head and neck cancer, intratumoral FDG uptake has been found to be associated with various tumor components (e.g., proliferating malignant cells, stromal tissue, and areas of necrosis) [35]. From a clinical standpoint, intratumoral heterogeneity as measured by ^18^F-FDG PET heterogeneity parameters (including homogeneity and entropy) has been proved to be superior to SUV for predicting response to chemoradiotherapy in patients with primary esophageal cancer [34]. In the current study, the heterogeneity parameter uniformity was an independent predictor of OS (HR = 1.977), whereas SUV, MTV, or TLG did not retain their independent significance in multivariate analysis. Uniformity represents the sum of squared elements in the gray-level co-occurrence matrix and is a measure of image homogeneity. The higher its value, the greater the image homogeneity [36]. ^18^F-FDG PET images characterized by low uniformity indicate that glucose metabolism is heterogeneous within the tumor, a feature with adverse prognostic and therapeutic implications [37]. Cook et al. [32] reported that ^18^F-FDG heterogeneity parameters contrast and entropy are independently associated with OS in non-small cell lung cancer (NSCLC). In addition, heterogeneity is an independent predictor of DSS (HR = 7.48) in NSCLC patients treated with stereotactic radiotherapy [30]. With regard to head and neck carcinoma, Cheng et al. [28] and Wang et al. [31] demonstrated that ^18^F-FDG PET texture uniformity independently predicted progression-free survival and disease-specific survival in patients with oropharyngeal carcinoma. In line with these findings, the results of our study indicate that uniformity is a significant prognostic factor for oropharyngeal and hypopharyngeal cancers. However, our current report differs from previous studies inasmuch as we included both oropharyngeal and hypopharyngeal cancers (*versus* oropharyngeal cancer only). In addition, published reports [28, 31] were retrospective reviews of clinical charts (which are prone to selection bias and inconsistencies in PET imaging protocols). Conversely, the current study was designed as a *post-hoc* analysis of prospectively collected data. Another strength of our study which was absent in previous investigations is the combination assessment of both DWI and DCE-MRI parameters. Unfortunately, we did not routinely assess human papillomavirus (HPV) markers in our study. Additional studies are required to investigate whether HPV data may further refine the prognostic stratification based on multiparametric imaging.

DCE-MRI allows the analysis of pharmacokinetic parameters in a selected tumor region and has been used to predict outcomes in HNSCC patients. K^*trans*^ reflects both tumor vascularity and permeability. Chawla et al. [12] reported that HNSCC patients who respond to chemoradiation have higher K^*trans*^ values than those who did not. It has been also demonstrated that HNSCC patients with higher pretreatment nodal K^*trans*^ values have a longer disease-free survival (*P* = 0.029) [38]. In the current study, K^*trans*^ retained its independent prognostic significance for both OS and RFS after allowance for potential confounders in multivariate analysis. These results emphasize the clinical usefulness of K^*trans*^ for predicting long-term outcomes in HNSCC patients. Because K^*trans*^ reflects blood flow and vascular permeability, it can serve as an index of lesion chemoradiosensitivity by acting as a proxy for the effective delivery of therapeutic agents and oxygen to the tumor. In contrast, K_*ep*_ reflects the backflow from the tumor to the vascular system and is related to tissue vascular permeability and the surface area. A higher K_*ep*_ implies a greater exchange of therapeutic agents between plasma and the extracellular extravascular space, facilitating drug delivery with resultant better treatment response. A previous DCE-MRI study by Coenegrachts et al. [39] demonstrated that K_ep_ is a significant predictor of treatment outcomes in patients with liver metastases from colorectal cancer. Zahra et al. [40] also showed that a higher K_*ep*_ is significantly correlated with the percentage tumor regression of cervical cancer after treatment with chemoradiotherapy. The association between K_*ep*_ and the prognosis of cancer patients remains unclear. In this study, we show that higher K_*ep*_ values independently predicted longer RFS (HR = 4.872) and OS (HR = 9.371) in OHSCC patients treated with chemoradiation.

DWI is a rapid MRI technique that allows to assess the diffusivity of water molecules in a tissue with ordered microstructure using the ADC [41]. DWI has been used to predict response to chemoradiotherapy in patients with HNSCC, albeit with conflicting results [6-12]. In our study, primary tumor ADC values were significantly associated with OS in univariate analysis; however, their independent prognostic value was not confirmed in multivariate analysis. Such discrepancies may at least in part be explained by the inclusion of different tumor types as well as distinct treatment protocols and clinical or imaging variables under investigation [37]. Notably, our current data confirm the results of a previous study showing that DWI is not prognostically useful in OHSCC patients [26].

**Figure 5:**
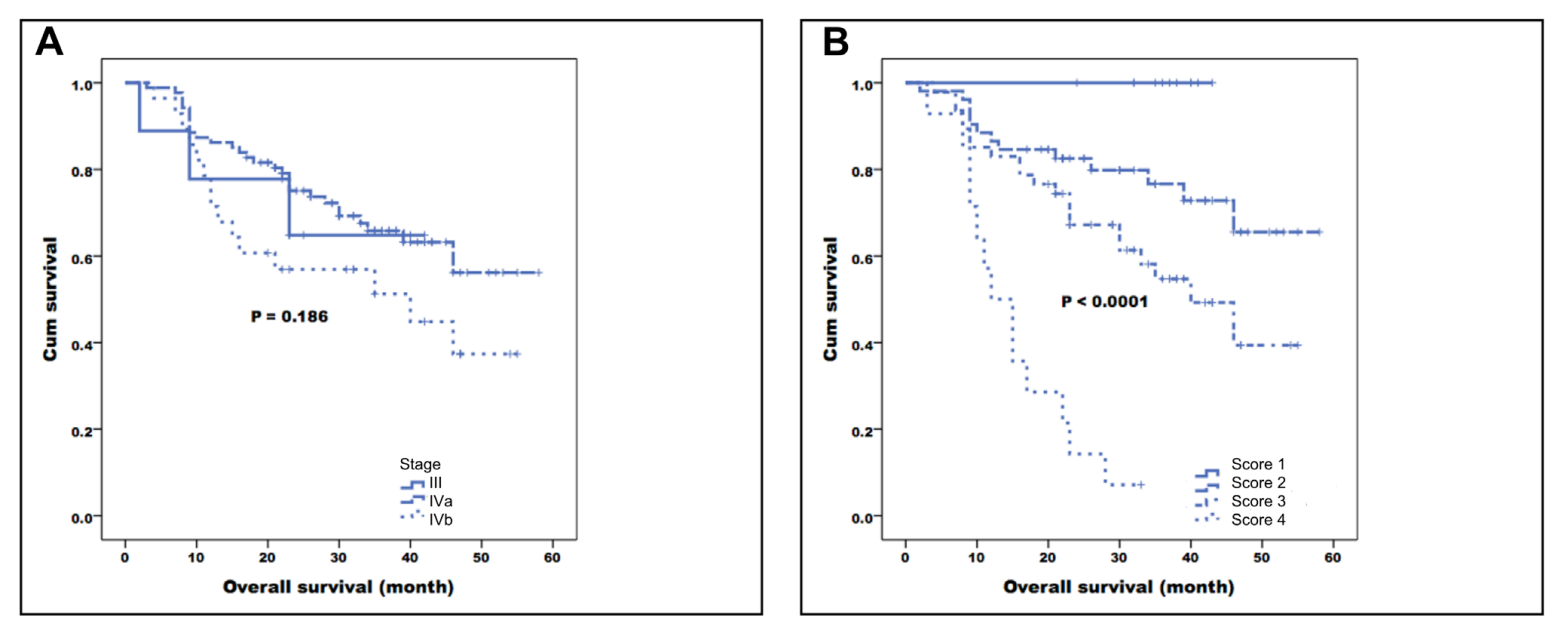
Kaplan-Meier plots of overall survival in patients with primary oropharyngeal or hypopharyngeal carcinoma stratified according to the TNM stage (**A**) and the independent risk factors (smoking, K^trans^, K_ep_, uniformity) identified in the current study **(B).** The combination of clinical variables, DCE-MRI, and PET heterogeneity parameters enabled a better prognostic stratification than the TNM staging system (*P* < 0.0001 *versus* 0.186).

The combined assessment of ^18^F-FDG PET heterogeneity and DCE-MRI parameters may shed more light on the metabolic and vascular phenotype of a malignancy. The associations between dynamic MRI parameters and heterogeneity on PET scans were modest in the current study, suggesting that they capture different biologic tumor features. Starting from these premises, we reasoned that their combined assessment could improve risk stratification of patients with primary OHSCC. To this aim, we formulated a prognostic scoring system based on the independent imaging and clinical variables identified in multivariate analysis. This system allowed to stratify patients into four groups (with 1, 2, 3, or 4 risk factors, respectively) that showed 3-year OS rates of 100%, 76.6%, 57.4%, and 7.1%, respectively (*P* < 0.0001). The scoring system identified in our current study was superior to both prognostic model derived from a previously published research [26] (modified with the exclusion of the nodal relative volume of extracellular extravascular space [V_*e*_-node]) based on DCE-MRI and conventional PET parameters and the traditional TNM staging system (Figures 2 and 5). The current prognostic stratification system may have significant clinical implications. It is well-known that intratumor heterogeneity is capable of fostering tumor adaptation and therapeutic resistance through natural selection [42]. However, the question as to whether the prediction accuracy of current prognostic models could be improved by the incorporation of PET heterogeneity parameters remains open. In addition, the potential advantage of combining ^18^F-FDG PET heterogeneity with functional MRI parameters in OHSCC patients has not been sufficiently explored. By measuring the intratumor differences in glucose metabolism or biological aggressiveness, we may be able to refine the prognostic stratification of OHSCC patients - a key step toward the delivery of specific treatments best suited for an individual under the framework of personalized medicine [43]. For example, patients with pharyngeal cancer and a favorable prognostic score may have the intensity of their induction chemotherapy or definitive CCRT lowered [44]. Notably, this approach has been recently validated in oropharyngeal cancer [45]. Such a strategy can in turn reduce long-term toxicity [46, 47] without compromising tumor control and/or survival. Our patients with a score of 1 showed a 3-year survival rate of 100.0%. We believe that such cases would be ideal candidates for less intensive treatment protocols in future prospective clinical trials. In contrast, patients with a score of 3 had a low-to-intermediate OS. Such cases could be suitable for trials of adjuvant chemotherapy aimed at reducing the likelihood of recurrent disease. Moreover, we recommend that patients with a score of 4 should undergo a strict surveillance protocol within the first two years after completion of chemoradiotherapy (for early detection of persistent or recurrent disease).

We have previously shown that DCE-MRI-, DWI-, and traditional PET-derived parameters predict prognosis in OHSCC patients [26]. When both conventional and heterogeneity PET parameters were examined in the current study, we identified a superior prognostic stratification in terms of OS stratification as compared with the previous report. Taken together, these data suggest that heterogeneity may further refine the prediction of prognosis in OHSCC patients.

Some caveats of our study merit consideration. First, DWI and DCE-MRI values depend on the choice of ROI. In addition, manual contouring of the tumor is an operator-dependent technique. All of the ROIs in the current study were drawn by an experienced head and neck radiologist to minimize potential biases. Second, it can be argued that the outcomes of chemoradiation may differ significantly between hypopharyngeal and oropharyngeal SCC, especially in presence of HPV-related tumors. Unfortunately, data on the HPV infection status were not available for this study. However, our OHSCC patients were treated more homogeneously than previous series focusing on different head and neck malignancies. Finally, a SUV of 2.5 was used for tumor contouring. This approach is in line with most previous studies focusing on PET parameters in patients with head and neck cancer [22, 28, 31, 48, 49], ultimately allowing a direct comparison with the published literature. The tumor volume defined by PET (MTV) was smaller than that measured on MRI (TVmri). The mean values (± standard deviations) of MTV and TVmri were 23.85 ± 23.80 mL and 37.92 ± 36.65 mL, respectively. The Spearman’s correlation rho value was 0.736, suggesting that MTV and TVmri had a marked reciprocal interrelation. The difference between MTV and TVmri may be attributed either to an underestimation on PET (resulting from our delineation method with fixed threshold or from a low FDG uptake related to a low tumor density at periphery) or an overestimation on MRI (caused by the presence of peritumoral inflammation). Unfortunately, we were unable to obtain pathological validation because our cases were treated by chemoradiation instead of surgery. Owing to the potential shortcomings of a fixed SUV threshold of 2.5, we also applied an adaptive threshold method for tumor segmentation. When the results obtained with the two methods were compared, the results of multivariate analysis with regard to recurrence-free survival were similar. Using the adaptive threshold method, we identified alcohol drinking, K^*trans*^, K_*ep*_, and uniformity as independent risk factors for overall survival. However, the resulting scoring system based on the combination of these parameters was not superior to that obtained when a fixed SUV threshold of 2.5 was used. There is no single widely accepted tumor segmentation method for PET imaging [50].

Another limitation of our study was that the limited number of our cases precluded validation in another cohort or cross-validation. Therefore, our study should be considered as hypothesis-generating. Indeed, the predictive capabilities of radiomic features should be thoroughly investigated with machine-learning approaches by the comparison of different feature selection and predictive modeling methods. Parmar et al. [51] have reported that the choice of classification method is the most dominant source of performance variation and identification of optimal machine-learning methods for radiomic applications facilitates the quantification of tumor-phenotypic characteristics in clinical practice. Nonetheless, further investigations are necessary to shed more light on the performance of different machine learning methods in PET images acquired from cancer patient

Quantization plays a critical role in feature repeatability and application. In this regard, a recent investigation by Leijenaar et al. demonstrated that patients ranked differently according to the feature values between intensity resampling methods; this could in turn hamper the clinical reliability of outcome prediction models [52]. The authors reported that maintaining a constant intensity resolution for SUV discretization across tumor images yielded textural feature values that are defined on the same SUV scale, allowing a meaningful comparison of texture features between images. In contrast, discretization of SUV values using a fixed number of bines was found to be less appropriate for inter- and intra-patient comparison of textural features in a clinical setting. Consequently, the modality of SUV discretization seems to have an important impact on the resulting textural features and should be taken into account in future studies.

In conclusion, the present study demonstrates that multiparametric imaging based on PET heterogeneity and DCE-MRI parameters in combination with clinical risk factors may improve the prognostic stratification of patients with advanced OHSCC treated with chemoradiation. It appeared to be superior compared to both the TNM staging system and the combination of the conventional PET parameters with functional MRI measures. Taken together, these results suggest that the combined assessment of different clinical, vascular, and metabolic heterogeneity parameters holds promise for refining the prognostic stratification of patients with malignancies.

## MATERIALS AND METHODS

### Study design

We performed a *post-hoc* analysis of data previously collected in a prospective investigation conducted at the Chang Gung Memorial Hospital, for which written informed consent was obtained from all participants. The study protocol (no. 98-3582B) followed the tenets of the Helsinki declaration and was approved by the Institutional Review Board of the Chang Gung Memorial Hospital.

### Patients

Consecutive patients with newly diagnosed OHSCC who were scheduled for chemoradiation with curative intent were deemed eligible. Inclusion criteria were as follows: (1) biopsy-proven OHSCC, (2) absence of distant metastases, (3) no contraindications to MRI or ^18^F-FDG PET/CT, and (4) ability to provide written informed consent. Patients with a history of previous head or neck malignancies, concomitant cancers in different anatomical districts, or renal failure were excluded. In the current study, we specifically focused on non-metastatic OHSCC patients with stage III-IV disease owing to their generally poor prognosis and absence of established prognostic factors.

### Definitive treatment and follow-up schedule

Patients were treated with intensity-modulated radiotherapy using 6-MV photon beams at 2 Grays (Gy) per fraction, with five fractions per week. Radiation therapy was delivered at a dose of 46 Gy to the gross tumor area (with at least 1-cm margins) and the entire neck, followed by a cone-down boost at 72 Gy to the gross tumor area and close margins. All participants received intensity-modulated radiotherapy. Concurrent chemotherapy was based on the administration of intravenous cisplatin 50 mg/m^2^ on day 1, oral tegafur 800 mg/day plus oral leucovorin 60 mg/day from day 1 to day 14. This scheme was repeated every two weeks throughout the radiotherapy course [53]. Following treatment, all patients underwent routine clinical follow-up examinations every 1 to 3 months. A follow-up MRI was performed at 3 months after completion of treatment. Subsequently, an additional MRI or CT scan was performed every 6 months or in presence of deteriorating clinical conditions. Patients were followed up for at least 12 months after treatment or until death.

### MRI and 18F-FDG PET/CT

All patients underwent MRI and ^18^F-FDG PET/CT before chemoradiotherapy. All of the examinations were performed within a time frame of 14 days before primary treatment. A 3-T MRI scanner (Magnetom Trio with TIM, Siemens, Erlangen, Germany) was used in this study. Conventional MRI images of the head and neck region were acquired in the axial and coronal projections using the following sequences: T1-weighted turbo spin echo TSE; T2-weighted TSE with fat saturation; and postcontrast fat-saturated T1-weighted TSE. Transverse images were obtained using a 5-mm section thickness. DWI was acquired using single shot spin-echo echo-planar imaging with a modified Stejskal-Tanner diffusion gradient pulsing scheme. Motion-probing gradients (b-value = 800 s/mm^2^) were applied along the three orthogonal directions. Imaging slices and coverage were identical for T1- and T2-weighted images. The repetition time (TR) and echo time (TE) were 8,200 ms and 84 ms, respectively. DCE-PWI was acquired using a 3D T1-weighted spoiled gradient-echo sequence with the following parameters: TR/TE = 3.5/1.13 ms, 230 × 230-mm field of view, and 108 × 128 matrix. The same imaging slice and coverage of conventional T1- and T2-weighted images were used. A spatial saturation slab was implanted below the acquired region to minimize the inflow effect from the carotid arteries. Before the administration of the contrast agent, baseline longitudinal relaxation time (T1_0_) values were calculated from images acquired with different flip angles (4°, 8°, 15°, and 25°). The dynamic series involved the use of the same sequence with a 15° flip angle. After four acquisitions of the dynamic baseline scanning, a standard dose (0.1 mmol/kg body weight) of gadopentetate dimeglumine (Gd-DTPA; Magnevist, Bayer-Schering, Burgess Hill, UK) was administered by a power injector through a cannula placed in the antecubital vein (rate = 3 mL/s) and immediately followed by a saline flush. A total of 80 volumes were acquired (temporal resolution = 3.3 s).

All of the study patients fasted for at least 6 h before ^18^F-FDG PET/CT imaging. Scans were performed with a PET/CT system (Discovery ST 16; GE Healthcare, Milwaukee, WI, USA) consisting of a PET scanner and a 16-section CT scanner. PET emission images were obtained between 50 and 70 min after injection of ^18^F-FDG (370 MBq) in the two-dimensional mode, with 3-min scanning time per table position. Before PET acquisition, a standardized helical CT scan was acquired from the head to the proximal thigh using the following settings: transverse 3.0-mm collimation × 16 modes, 100 kVp, 100 mAs, 0.5-s tube rotation, 35-mm/s table speed, and 1.5 pitch. No intravenous iodinated contrast agent was used. CT data were resized from a 512 × 512 matrix to a 128 × 128 matrix to match PET results and generate fused images and CT-based transmission maps. PET images were reconstructed with CT for attenuation correction and an ordered-subset expectation maximization iterative reconstruction algorithm (4 iterations and 10 subsets).

### Image analysis

Tumor segmentation in PET images was performed with the PMOD 3.2 software package (PMOD Technologies Ltd, Zurich, Switzerland). First, boundaries were drawn by an experienced nuclear medicine physician (blinded to clinical data) largely enough to include the primary tumor in the axial, coronal, and sagittal 18F-FDG PET scans. A joint reading of both head and neck MRI and 18F-FDG PET scans was performed side-by-side to avoid the inclusion of areas with physiological FDG uptake within the regions of interest. The volumes of interest (VOIs) were checked and validated by an independent senior nuclear medicine physician. Second, the tumor boundaries were defined using a fixed SUV threshold of 2.5 and with an adaptive threshold method [54], respectively. The background of the adaptive threshold was determined as the mean uptake of a 10-mm VOI sphere placed in the lower neck where no lymph node lesions were evident in the study subjects. The voxels presenting a SUV intensity >2.5 or greater than the adaptive threshold within the contouring margin were incorporated for MTV determination. Finally, SUV (maximal, mean), MTV, and TLG of the lesion were automatically calculated by the software as previously described [55]. The core data discussed in the study were based on tumor segmentation using a fixed SUV threshold of 2.5. The results obtained by applying the adaptive threshold method were presented separately and in the Supplementary Data.

Heterogeneity parameters or texture features were determined using the normalized gray-level co-occurrence matrix (NGLCM) and the neighborhood gray-tone difference matrix (NGTDM). First, voxel intensities were resampled within the segmented tumors to yield a limited range of values, with the ultimate goal of reducing noise and normalizing images. The intensity of ^18^F-FDG uptake in the primary tumor was resampled to both 32 and 64 different values. Subsequently, the texture features were calculated as reported previously [28]. Second-order textural features were calculated using the normalized grey-level co-occurrence matrix (NGLCM), which indicates how frequently a voxel of resampled intensity i is neighbor to another voxel of intensity j; it includes the parameters of uniformity, entropy, dissimilarity, contrast, homogeneity, inverse different moment, and correlation. We considered all of the 13 possible space directions to determine NGLCM-based parameters and the resulting values from all directions are averaged. The three-dimensional NGLCM was applied in an orientation-invariant manner for calculation of all parameters. Higher-order parameters were calculated using neighborhood intensity difference matrices (NGTDM) to describe the local features. The NGTDM are based on the differences between each voxel and the neighboring voxels in the adjacent image planes. Texture parameters derived from NGTDM resemble the human perception of the image. After image intensity resampling, we calculated coarseness, contrast, busyness, complexity, and strength. All of the texture features were calculated using an in-house software package (Chang-Gung Image Texture Analysis toolbox, CGITA) implemented under MATLAB 2012a (Mathworks Inc., Natick, MA, USA).

DWI-derived apparent diffusion coefficient (ADC) values were measured on ADC maps by drawing the regions of interest (ROI) on the primary tumors by an experienced head and neck radiologist, with the aid of T2-weighted MRI images and the T1-weighted post-contrast MRI images. DCE-MRI analysis was performed using MATLAB 7.0 (The Mathworks, Natick, MA, USA). The extended Kety model [15] was used in a voxel-wise manner for the pharmacokinetic analysis. The arterial input function was extracted using the blind source separation algorithm [56]. ROIs were manually drawn on DCE-MRI images by the same head and neck radiologist. The following pharmacokinetic parameters were collected: the volume transfer rate constant (K^*trans*^), relative extravascular extracellular space (V_*e*_) and relative vascular plasma volume (V_*p*_) as well as the efflux rate constant (K_*ep*_) which equals the ratio of K^*trans*^ to V_*e*_.

### Outcome determination and statistical analysis

All of the patients were followed up until August 2015 or death. OS and RFS served as the main outcome measures. OS was calculated from the date of diagnosis to the date of death or censored at the date of the last follow-up for surviving patients. RFS was defined as the time between the end of treatment and the date of recurrence (tumor relapse or death) or censored at the date of the last follow-up. The cutoff values for the variables were determined using the log-rank test based on the RFS and OS rates observed in the entire study cohort [21, 57]. Cox regression models were used to identify the predictors of survival outcomes. The effect of each individual variable was initially investigated using univariate analysis. Multivariate regression models were then constructed to identify the independent predictors of survival after allowance for potential confounders. Survival curves were plotted using the Kaplan-Meier method. The associations between clinical variables and DCE-MRI or PET parameters were examined with the Mann-Whitney *U* test to compare the differences of these parameters in different clinical groups. Two-tailed *p* values < 0.05 were considered statistically significant.

We also compared the prognostic system developed in the current study with a scoring method described in a previous paper from our group [26]. The latter system was solely based on conventional PET parameters and functional MRI measures. The current study cohort (n=124) includes 86 patients already examined in the previous report. For the purpose of comparison with the currently developed system, the previous model has been modified to include K_*ep*_ and SUV of the primary tumor. We excluded the relative volume of the extracellular extravascular space of the neck metastatic node [V_*e*_-node], which cannot be assessed in patients with N0 disease enrolled in the current study.

## SUPPLEMENTARY MATERIALS FIGURE AND TABLES


