# Correction: Multiparametric imaging using ^18^F-FDG PET/CT heterogeneity parameters and functional MRI techniques: prognostic significance in patients with primary advanced oropharyngeal or hypopharyngeal squamous cell carcinoma treated with chemoradiotherapy

**DOI:** 10.18632/oncotarget.24463

**Published:** 2018-02-09

**Authors:** Sheng-Chieh Chan, Nai-Ming Cheng, Chia-Hsun Hsieh, Shu-Hang Ng, Chien-Yu Lin, Tzu-Chen Yen, Cheng-Lung Hsu, Hung-Ming Wan, Chun-Ta Liao, Kai-Ping Chang, Jiun-Jie Wang

**Affiliations:** ^1^ Department of Nuclear Medicine, Keelung Chang Gung Memorial Hospital, Keelung, Taiwan; ^2^ Molecular Imaging Center, Linkou Chang Gung Memorial Hospital and Chang Gung University, Taoyuan, Taiwan; ^3^ Department of Nuclear Medicine, Linkou Chang Gung Memorial Hospital and Chang Gung University, Taoyuan, Taiwan; ^4^ Department of Internal Medicine, Division of Medical Oncology, Linkou Chang Gung Memorial Hospital and Chang Gung University, Taoyuan, Taiwan; ^5^ Department of Diagnostic Radiology, Linkou Chang Gung Memorial Hospital and Chang Gung University, Taoyuan, Taiwan; ^6^ Department of Medical Imaging and Radiological Sciences, Chang Gung University, Taoyuan, Taiwan; ^7^ Department of Radiation Oncology, Linkou Chang Gung Memorial Hospital and Chang Gung University, Taoyuan, Taiwan; ^8^ Department of Otorhinolaryngology, Linkou Chang Gung Memorial Hospital and Chang Gung University, Taoyuan, Taiwan

**This article has been corrected:** the 5^th^ affiliation citation has been included to Dr. Jiun-Jie Wang along with affiliation 6^th^.

Original article: Oncotarget. 2017; 8:62606-62621. https://doi.org/10.18632/oncotarget.15904

