# Supplementary material for: Multiparametric imaging using 18F-FDG PET/CT heterogeneity parameters and functional MRI techniques: prognostic significance in patients with primary advanced oropharyngeal or hypopharyngeal squamous cell carcinoma treated with chemoradiotherapy
Source: Oncotarget. 2017 Mar 4;8(37):62606–21. doi: 10.18632/oncotarget.15904 (PMC5617533; doi:10.18632/oncotarget.15904)
Supplement: Supplementary file 1 [file oncotarget-08-62606-s001.pdf]

Multiparametric imaging using ^{18}F-FDG PET/CT heterogeneity parameters and functional MRI techniques: prognostic significance in patients with primary advanced oropharyngeal or hypopharyngeal squamous cell carcinoma treated with chemoradiotherapy

Supplementary Materials

Supplementary Table 1. Recurrence-free survival rates stratified according to the prognostic scoring system

	P value	HR (95% CI)
Score 1 (n = 20)	0.0006	0.071 (0.016−0.322)
Score 2 (n = 35)	0.0003	0.197 (0.081−0.480)
Score 3 (n = 54)	0.011	0.406 (0.204−0.811)
Score 4 (n = 15)	Reference	Reference

Abbreviations: HR, hazard ratio; CI, confidence interval.

Supplementary Table 2. Association of clinical variables with DCE-MRI and PET heterogeneity parameters

	No.	K^{trans}		K_{ep}		Uniformity	
		Mean	*P* value*	Mean	*P* value	Mean	*P* value
Sex			0.927		0.371		0.776
Male	116	0.531304		3.401956		0.002710	
Female	8	0.564563		2.765613		0.002086	
Age, years			0.163		0.104		0.256
≤ 50	63	0.571295		3.713102		0.002446	
> 50	61	0.494364		2.997154		0.002901	
Tumor subsite			0.052		0.591		0.619
Oropharynx	63	0.579538		3.486744		0.002735	
Hypopharynx	61	0.485851		3.230933		0.002602	
T classification			0.639		0.427		0.0001
T1-3	51	0.562900		3.633769		0.003391	
T4	73	0.512875		3.170268		0.002165	
N classification			0.607		0.186		0.283
N0-1	27	0.492204		2.868141		0.001961	
N2-3	97	0.544931		3.498062		0.002867	
Tumor stage			0.705		0.357		0.006
III	9	0.486011		3.379233		0.002435	
IIIA	87	0.556828		3.567614		0.003028	
IIIB	28	0.476061		2.712725		0.001632	
Smoking			0.879		0.521		0.388
Yes	94	0.531515		3.548496		0.002469	
No	30	0.539513		2.773107		0.003299	
Alcohol drinking			0.670		0.534		0.059
Yes	80	0.536371		3.260981		0.002367	
No	44	0.528139		3.542575		0.003219	
Betel quid chewing			0.018		0.621		0.355
Yes	62	0.592350		3.480431		0.002113	
No	62	0.474550		3.241373		0.003226	
Hemoglobin (g/dL)			0.786		0.657		0.111
≤14.3	73	0.534220		3.339466		0.002857	
>14.3	51	0.532452		3.388689		0.002426	

* Mann-Whitney *U* test

Supplementary Table 3. Correlations of conventional ^{18}F-FDG PET parameters with DCE-MRI and PET heterogeneity parameters which were identified as independent risk factors

	Uniformity	K^{trans}	K_{ep}
TLG<br>(g/mL × mL)	ρ=-0.395<br>*P*<0.001	ρ=0.044<br>*P*=0.629	ρ=0.008<br>*P*=0.931
MTV<br>(mL)	ρ=-0.343<br>*P*<0.001	ρ=0.095<br>*P*=0.296	ρ=0.045<br>*P*=0.616
SUV<br>(g/mL)	ρ=-0.086<br>*P*=0.340	ρ=-0.132<br>*P*=0.144	ρ=-0.062<br>*P*=0.493

Supplementary Table 4. Multivariate analysis of risk factors in relation to overall survival according to different segmentation methods

Risk factor	Fixed SUV threshold of 2.5		Adaptive threshold	
	HR (95% CI)	*P* value	HR (95% CI)	*P* value
Clinical variable				
Tumor subsite	-	N/A	-	N/A
T status	-	N/A	-	N/A
Smoking	2.519 (1.264−5.019)	0.009	-	Ns
Alcohol drinking	-	Ns	2.394 (1.198−4.786)	0.013
DCE-MRI and DWI parameters				
Ktrans	3.648 (1.843−7.223)	0.0002	4.116 (2.143−7.905)	<0.0001
Vp	-	Ns	-	Ns
Ve	-	Ns	-	Ns
Kep	9.731 (2.046−42.919)	0.004	9.866 (2.342−41.557)	0.002
ADC	-	Ns		Ns
PET/CT parameters				
SUV	-	N/A	-	N/A
MTV	-	Ns	-	Ns
TLG	-	N/A	-	N/A
Uniformity	1.977 (1.075−3.636)	0.028	1.996 (1.100−3.662)	0.023

Abbreviations: OS, overall survival; RFS, recurrence-free survival; HR, hazard ratio; CI, confidence interval; EBV, Epstein-Barr virus; NS, non-significant; N/A, not applicable.

Supplementary Figure 1

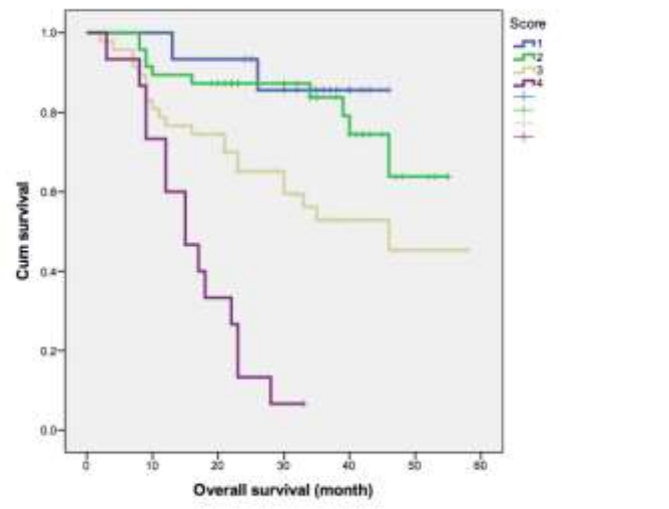	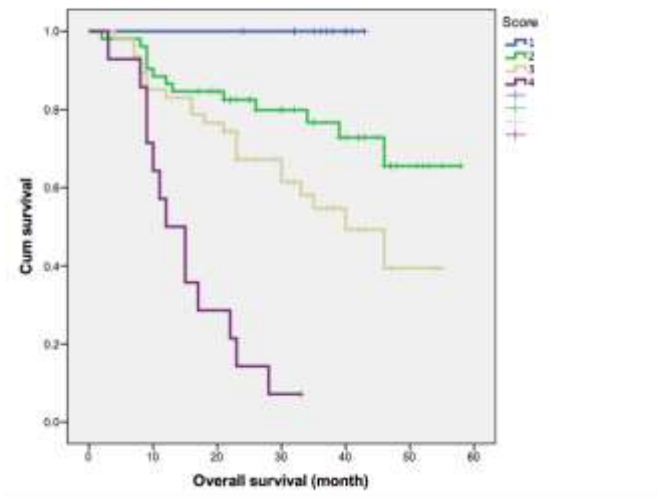
Overall survival curves obtained when the adaptive threshold method was used for segmentation.	Overall survival curves obtained when a fixed SUV threshold of 2.5 was used for segmentation.

Supplementary Table 5. Multivariate analysis of risk factors in relation to recurrence-free survival according to different segmentation methods

Risk factor	Fixed SUV threshold of 2.5		Adaptive threshold	
	HR (95% CI)	*P* value	HR (95% CI)	*P* value
Clinical variable				
Tumor subsite	1.957 (1.037−3.692)	0.038	2.155 (1.174−3.957)	0.013
T status	-	Ns	-	Ns
Smoking	-	N/A	-	N/A
Alcohol drinking	2.840 (1.349−5.979)	0.006	2.492 (1.189−5.224)	0.016
DCE-MRI and DWI parameters				
Ktrans	2.373 (1.164−4.840)	0.017	2.511 (1.368−4.608)	0.003
Vp	-	Ns	-	Ns
Ve	-	Ns	-	Ns
Kep	4.872 (1.594−14.888)	0.005	4.081 (1.547−10.763)	0.004
ADC	-	N/A	-	N/A
PET/CT parameters				
SUV	-	Ns	-	Ns
MTV	-	Ns	-	Ns
TLG	-	Ns	-	Ns

Uniformity	-	N/A	-	N/A

Abbreviations: OS, overall survival; RFS, recurrence-free survival; HR, hazard ratio; CI, confidence interval; EBV, Epstein-Barr virus; NS, non-significant; N/A, not applicable.

Multimodal imaging and clinical parameters in relation to treatment response

Of the 124 OHSCC patients, 93 (75%) achieved complete remission 3 months after definitive chemoradiotherapy. Multimodal imaging and clinical parameters were examined in relation to treatment response (Supplementary Table 6). Univariate analysis revealed that V_e was the only variable significantly associated with treatment response (P = 0.022). K^{trans}, K_{ep}, uniformity, and other clinical variables did not show statistically significant associations.

Supplementary Table 6. Multimodal imaging and clinical parameters in relation to treatment response in patients with primary oropharyngeal/hypopharyngeal cancer (n = 124)

Risk factor (n)	Univariate analysis	
	No. of non-CR	*P* value
Age (years)		0.410
≤ 50 (63)	18	
> 50 (61)	13	
Sex		0.359
Male (116)	30	
Female (8)	1	
Tumor subsite		0.062
Oropharynx (63)	11	
Hypopharynx (61)	20	
Hemoglobin (g/dL)		0.676
≤14.3 (73)	19	
>14.3 (51)	12	
T status		0.058
T1-3 (51)	8	
T4 (73)	23	
N status		0.458
N0-1 (27)	5	
N2-3 (97)	26	
Disease stage		0.139
III (9)	2	
IVA (87)	18	
IVB (28)	11	
Smoking		0.167
Yes (94)	5	
No (30)	26	
Alcohol drinking		0.139
Yes (80)	8	
No (44)	23	
Betel quid chewing		0.679
Yes (62)	14	
No (62)	17	
K^{trans}		0.097
≤ 0.5512 (68)	21	
> 0.5512 (56)	10	
V_p × 1000		0.794
≤ 0.0451 (24)	5	
> 0.0451 (100)	26	
V_e		0.022
≤ 0.3399 (98)	29	
> 0.3399 (26)	2	
K_{ep}		0.398
≤ 0.8872 (20)	3	
> 0.8872 (104)	28	
ADC		0.589
≤ 836.2 (22)	4	
> 836.2 (102)	27	
SUV		0.064
≤ 14.22 (54)	9	
> 14.22 (70)	22	
MTV		0.089
≤ 24.01 (80)	16	

> 24.01 (44)	15	
TLG		0.735
≤ 408.534 (111)	27	
> 408.534 (13)	4	
Uniformity		0.201
≤ 0.00381 (47)	15	
> 0.00381 (77)	16	

Abbreviations: CR, complete response; ADC, apparent diffusion coefficient; SUV, standardized uptake value; MTV, metabolic tumor volume; TLG, total lesion glycolysis. *P* values were calculated with the Fisher's exact test.
